# Addendum: Thermal sensitivity of CO_2_ and CH_4_ emissions varies with streambed sediment properties

**DOI:** 10.1038/s41467-019-11185-x

**Published:** 2019-07-09

**Authors:** Sophie A. Comer-Warner, Paul Romeijn, Daren C. Gooddy, Sami Ullah, Nicholas Kettridge, Benjamin Marchant, David M. Hannah, Stefan Krause

**Affiliations:** 1 School of Geography, Earth and Environmental Sciences, University of Birmingham, Edgbaston, Birmingham, B15 2TT UK; 2 British Geological Survey, Maclean Building, Wallingford, Oxfordshire OX10 8BB UK

Addendum to: *Nature Communications* 10.1038/s41467-018-04756-x; published online 18 July 2018.

We would like to clarify the discussion in this Article of the previously published relationships between temperature and greenhouse gas (GHG) emissions. Previous studies identified complex relationships between temperature and GHG emissions or microbial metabolic activity, some of them pointing towards exponential behaviour following Arrhenius law^1–3^. The results of the study presented in this Article specifically highlight non-linearity in the temperature dependency of GHG emissions, which was not exponential. This includes threshold responses of streambed GHG production as a function of streambed warming, with temperature sensitivity varying greatly with substrate, organic matter content, and geological origin. Our results, therefore, demonstrate the relevance of global warming impacts on streambed GHG production; especially due to observed non-linearity in streambed GHG production with increased temperature.
