# Supplementary material for: Thermal sensitivity of CO2 and CH4 emissions varies with streambed sediment properties
Source: Nat Commun. 2018 Jul 18;9:2803. doi: 10.1038/s41467-018-04756-x (PMC6052154; doi:10.1038/s41467-018-04756-x)
Supplement: Supplementary file 1 — Supplementary Information [file 41467_2018_4756_MOESM1_ESM.pdf]

Supplementary Information

Thermal sensitivity of CO_2 and CH_4 emissions varies with streambed sediment properties

Comer-Warner et al.

Supplementary Tables

Contrast	Intersect Estimate	p-value	Gradient Estimate	p-value	Mean by jar Estimate	p-value
ANOVA		**<0.01**		**<0.01**		**<0.01**
C1	-0.13	0.83	-0.16	**<0.01**	-3.46	**<0.01**
C2	1.16	**0.02**	0.01	0.65	1.39	**<0.01**
C3	3.28	**<0.01**	-0.04	0.37	2.13	**<0.01**
C4	2.11	**<0.01**	0.00	0.95	1.94	**<0.01**
C5	-1.57	0.13	-0.29	**<0.01**	-7.32	**<0.01**

Supplementary Table 1. Statistical analysis results for MMA. Estimated value of each intersect, gradient and jar-mean contrast and the probability of these estimates being achieved under the null model Ci=0 for $log(Rru + 0.1)$. *Significant p-values are in bold.*

**Temperature (°C)**	**Substrate**	Q_{10MMA}	Q_{10CO2}	Q_{10CH4}
5 and 9	$Chalk_{fine}$	2.1	0.3	2.6
5 and 9	$Chalk_{medium}$	0.0	0.1	0.6
5 and 9	$Chalk_{coarse}$	0.0	1.1	0.0
5 and 9	$Sandstone_{fine}$	0.0	0.6	421.1
5 and 9	$Sandstone_{medium}$	0.0	0.5	-
5 and 9	$Sandstone_{coarse}$	-	3.5	-
5 and 9	Control	-	1.0	-
9 and 15	$Chalk_{fine}$	9.0	8.1	134.9
9 and 15	$Chalk_{medium}$	-	80.6	115.6
9 and 15	$Chalk_{coarse}$	-	0.1	9.5
9 and 15	$Sandstone_{fine}$	-	2.7	227.6
9 and 15	$Sandstone_{medium}$	-	4.0	7.2
9 and 15	$Sandstone_{coarse}$	-	9.1	0.9
9 and 15	Control	-	0.7	1.0
15 and 21	$Chalk_{fine}$	22.2	1.4	9.3
15 and 21	$Chalk_{medium}$	1425.3	2.3	163.2
15 and 21	$Chalk_{coarse}$	13.0	3505.2	78.8
15 and 21	$Sandstone_{fine}$	3.3	0.8	4.1
15 and 21	$Sandstone_{medium}$	52.6	1.8	4.1
15 and 21	$Sandstone_{coarse}$	65.9	1.0	10.1
15 and 21	Control	-	0.2	-
21 and 26	$Chalk_{fine}$	0.9	2.3	0.1
21 and 26	$Chalk_{medium}$	0.2	1.5	0.0
21 and 26	$Chalk_{coarse}$	2.0	100.3	0.0
21 and 26	$Sandstone_{fine}$	2.0	4.9	12.2
21 and 26	$Sandstone_{medium}$	2.3	4.6	0.1
21 and 26	$Sandstone_{coarse}$	2.3	4.0	10.1
21 and 26	Control	-	0.6	-

Supplementary Table 2. Temperature coefficient (Q_{10}) values of CO_2, CH_4 and MMA with temperature increase. In some cases where production was negative or zero, no Q_{10} values could be calculated.

	Intersect		Gradient		Mean by jar	
Contrast	Estimate	p-value	Estimate	p-value	Estimate	p-value
ANOVA		**<0.01**		**<0.01**		**<0.01**
C1	-1.82	0.6	-1.01	**<0.01**	-17.23	**<0.01**
C2	-6.01	**0.02**	0.46	**<0.01**	0.87	0.25
C3	10.59	**0.01**	1.47	**<0.01**	32.98	**<0.01**
C4	12.93	**<0.01**	0.65	**<0.01**	22.55	**<0.01**
C5	-15.79	**<0.01**	-3.57	**<0.01**	-70.01	**<0.01**

Supplementary Table 3. Statistical analysis results for CO_2. Estimated value of each intersect, gradient and jar-mean contrast and the probability of these estimates being achieved under the null model Ci=0 for CO_2. Significant p-values are in bold.

	Intersect		Gradient		Mean by jar	
Contrast	Estimate	p-value	Estimate	p-value	Estimate	p-value
ANOVA		**<0.01**		**<0.01**		**<0.01**
C1	-4.65	0.76	-0.08	**<0.01**	-1.73	**<0.01**
C2	-3.05	**0.01**	0.06	**0.46**	1.30	**<0.01**
C3	-4.60	**<0.01**	0.16	**<0.01**	2.89	**<0.01**
C4	-4.71	**0.22**	0.14	**<0.01**	2.67	**<0.01**
C5	-5.31	**<0.01**	-0.36	**<0.01**	-2.58	**<0.01**

Supplementary Table 4. Statistical analysis results for CH_4. Estimated value of each intersect, gradient and jar-mean contrast and probability of these estimate being achieved under the null model Ci=0 for $log(CH_4 + 0.01)$. Significant p-values are in bold.

Contrast Number	Contrast Equation	Contrast Comparison
C1	$\alpha_0 - \frac{1}{6}\sum_{i=1}^{6}\alpha_i$	The intersect of the control to the average of the six sediment types
C2	$\frac{1}{3}\sum_{i=1}^{3}\alpha_i - \frac{1}{3}\sum_{i=4}^{6}\alpha_i$	The intersects for Chalk to those for Sandstone
C3	$\alpha_1 - \frac{\alpha_2 + \alpha_3}{2}$	The intersect for $Chalk_{fine}$ to the average of that for the other Chalk classes
C4	$\alpha_4 - \frac{\alpha_5 + \alpha_6}{2}$	The intersect for $Sandstone_{fine}$ to the average of that for the other Sandstone classes
C5	$\alpha_0 - \frac{\alpha_1 + \alpha_4}{2}$	The intersect of the control to the average of those for fine sediment classes

Supplementary Table 5. Details of the statistical comparisons performed. The orthogonal contrasts investigated to determine the statistical significance of the effect of temperature and sediment type on MMA and GHG production.

Supplementary Figures

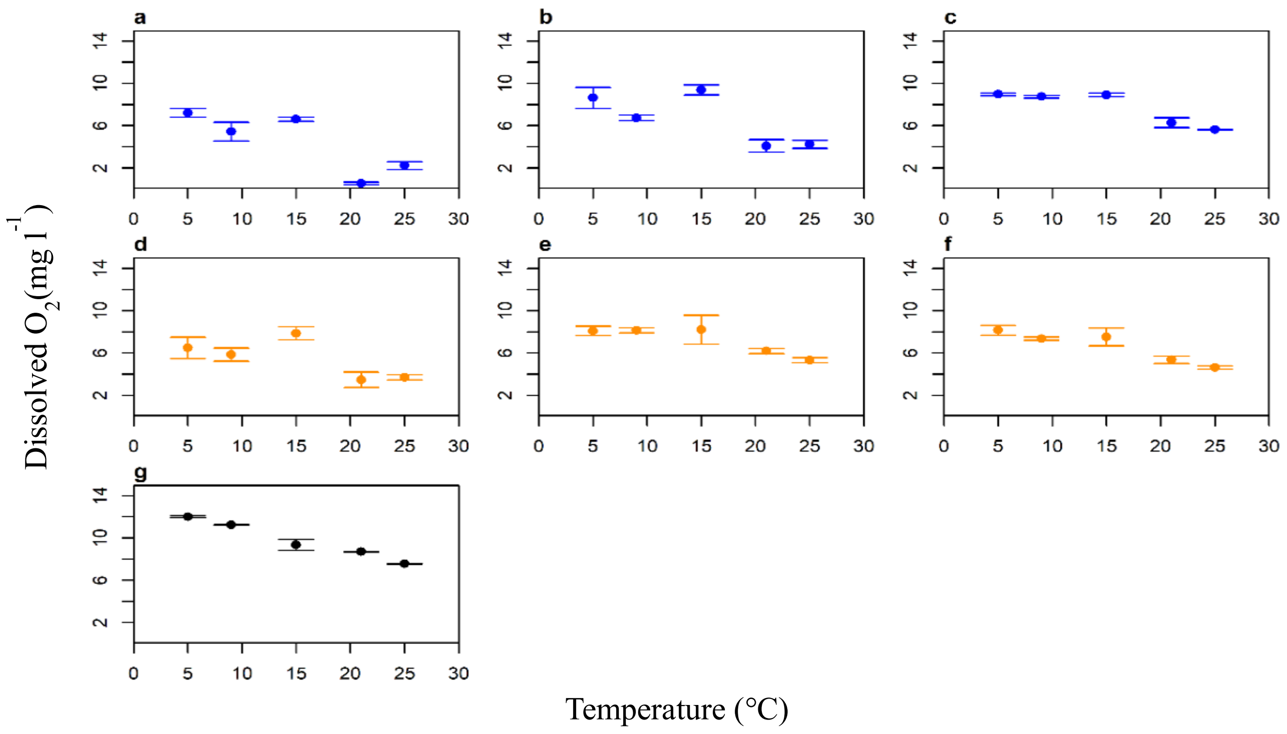

Supplementary Figure 1. Dissolved oxygen concentrations for each sediment type over the temperature range of the incubation experiments. Dissolved oxygen concentration plotted against temperature for a – Chalk$_{fine}$, b – Chalk$_{medium}$, c – Chalk$_{coarse}$, d – Sandstone$_{fine}$, e – Sandstone$_{medium}$, f – Sandstone$_{coarse}$ and g – Control. Chalk results are shown in blue, Sandstone in orange, and controls in black. The error bars represent one standard deviation.

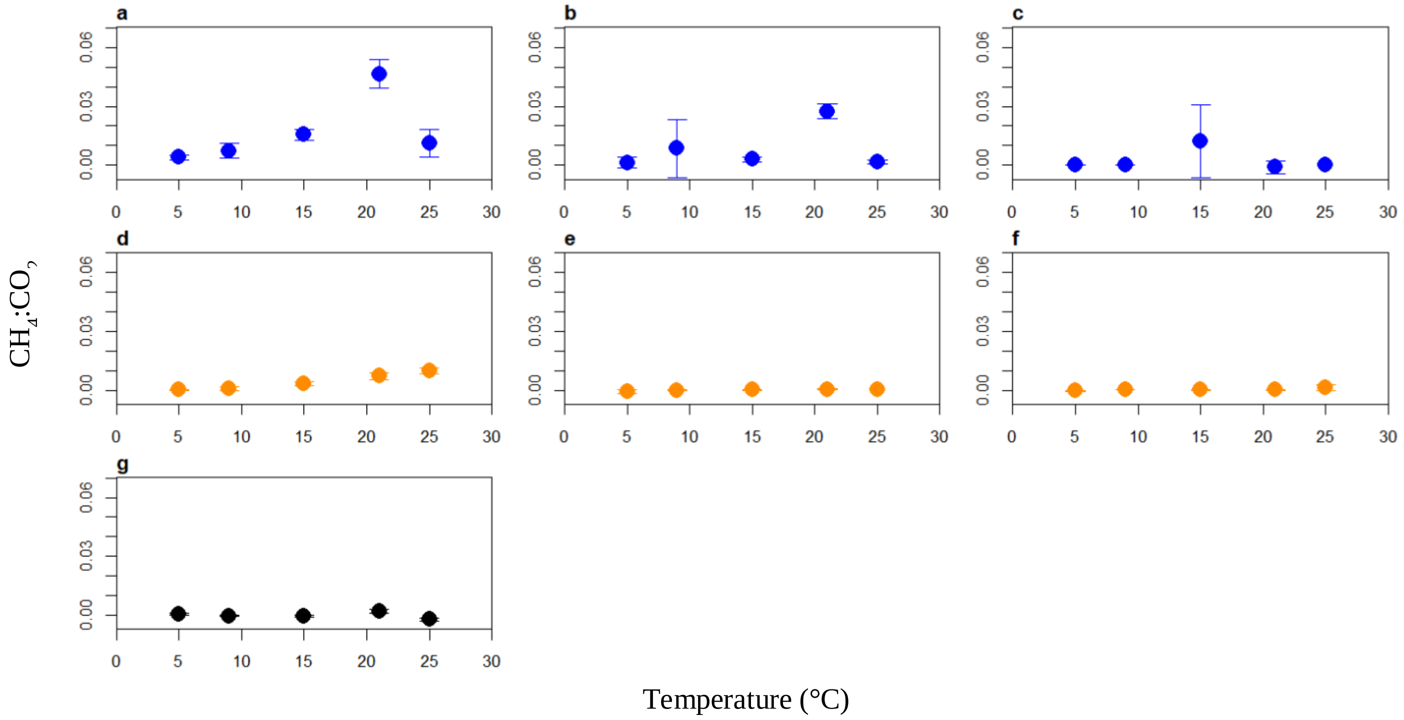

Supplementary Figure 2. The ratios of methane to carbon dioxide for each sediment type over the temperature range of the incubation experiments. CH_4:CO_2 ratios plotted against temperature for a – Chalk$_{fine}$, b – Chalk$_{medium}$, c – Chalk$_{coarse}$, d – Sandstone$_{fine}$, e – Sandstone$_{medium}$, f – Sandstone$_{coarse}$ and g – Control. Chalk results are shown in blue, Sandstone in orange, and controls in black. The error bars represent one standard deviation.

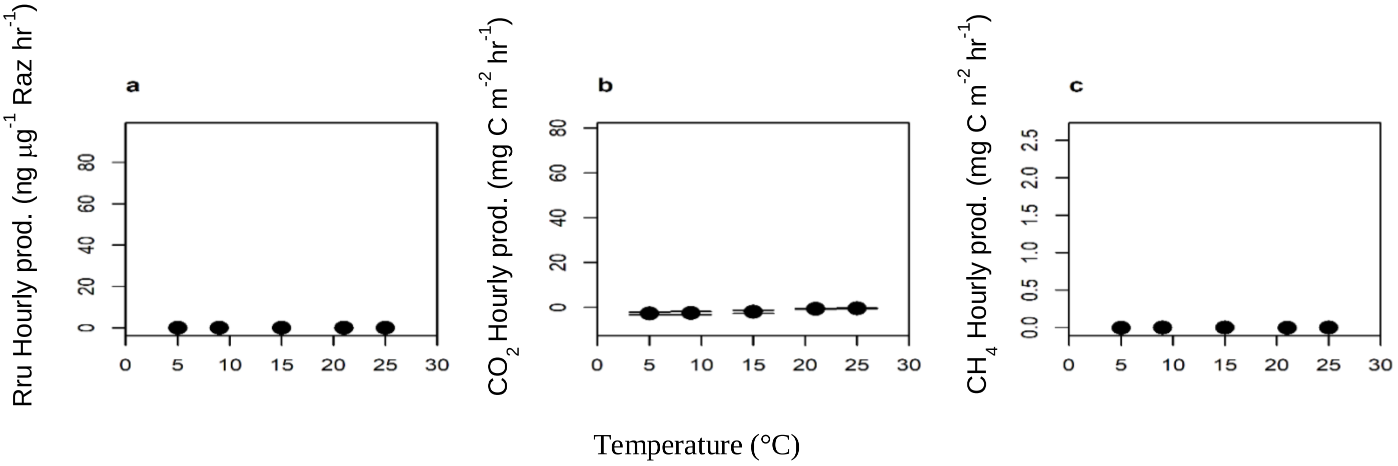

Supplementary Figure 3. Hourly production of MMA and GHG production for the control experiments over the range of temperature treatments. Hourly production plotted against temperature for control experiments. a Rru production, b CO_2 production and c CH_4 production. The error bars represent one standard deviation.
